# Combatting Antibiotic Resistance Together: How Can We Enlist the Help of Industry?

**DOI:** 10.3390/antibiotics7040111

**Published:** 2018-12-18

**Authors:** Suzanne E. Edwards, Chantal M. Morel, Reinhard Busse, Stephan Harbarth

**Affiliations:** 1 Department of Health Care Management, Berlin University of Technology, Straße des 17. Juni 135, 10623 Berlin, Germany; esuzi@hotmail.co.uk (S.E.E.); rbusse@tu-berlin.de (R.B.); 2 Infection Control Programme, WHO Collaborating Centre, University of Geneva Hospitals and Faculty of Medicine, Rue Gabrielle-Perret-Gentil 4, 1205 Geneva, Switzerland; stephan.harbarth@hcuge.ch; 3 LSE Health, London School of Economics and Political Science, Houghton Street, London WC2A 2AE, UK

**Keywords:** antibiotics, antimicrobial resistance, antibiotic consumption, marketing, promotion, pharmaceutical industry, research and development incentives, health economics, sustainable use

## Abstract

The development of antibiotics needs to be supported through new financial stimuli, including help from the public sector. In exchange for public support, industry should be asked to do what is in their power to help curb the inappropriate use of antibiotics. This work discusses key areas through which industry has an important influence on antibiotic consumption and where agreements can be made alongside financial incentives, even those intended to stimulate very early research. As long as the traditional unit sale-based business model for antibiotics remains in place, profit-making incentives will likely undermine efforts to sell and utilize antibiotics in a sustainable manner. In the short-term, while we try to come to a consensus on how best to fix the market, we need measures to prevent major over-selling and inappropriate promotion—especially for new, badly needed antibiotics that reach the market. This paper explores ways in which the pharmaceutical industry could help buttress sustainable antibiotic use while we search for more long-term, constructive, mutually-beneficial ways to organize the market.

## 1. Introduction

The rapid growth of antibiotic resistance and the slow arrival of new, innovative, anti-infective agents on the market, are now at the forefront of public debate. There is a sense of urgency to address the problem in the highest political and economic fora and a recognition that doing so requires engagement from a broad array of actors—including governments, regulators, experts in health, environment, economics, trade, and industry [1]. Repeatedly on the agenda is the need to find incentives to re-ignite research and development (R&D) for new antibiotics. Several studies have analyzed the economics of doing so through a variety of approaches [2,3]. The sums being considered are immense, with potential rewards of up to two billion dollars for bringing a product to market. This work considers how drug developers could be asked to join the fight against antibiotic resistance when accepting one of these large-scale R&D rewards. It is an exploration of how pharmaceutical companies could utilize their global presence and their ability to influence sales and consumption to foster the sustainable use (SU) of antibiotics when accepting large public subsidies, regardless of the precise ways in which the subsidies are designed.

## 2. Background: Current Responses Are Insufficient

Thus far, governments have responded to the lack of innovation in antibiotic research with a combination of push funding (that lowers the barrier to market entry) and public-private collaboration (which pools expertise). Notable pre-market examples include the COMBACTE network in Europe [4], Carb-X [5], and the GARDP initiative [6]. One analysis suggests that 50% of current antibiotics in development are being done so in collaboration [7]. Post-market efforts have focused on supporting surveillance, through initiatives such as EPI-Net and the recent Wellcome Trust/Open Data Institute (ODI) pilot. However, alone, these efforts will be insufficient to bring new products to market—a convincing ‘pull incentive’ will also be required to lure antibiotic candidates through to licensure. Such large-scale rewards provide the opportunity to change how the market functions. Indeed, the need for public subsidization combined with the inconvenient reality of resistance development over time, precludes antibiotics from being considered traditional goods that can be mass marketed by suppliers. Several proposals for pull incentives have been put on the table, which alter the underlying business model in order to remove perverse incentives and hedge against superfluous antibiotic sales (e.g., by delinking unit sales from R&D revenues through large extra-market rewards).

In response to the proposed overhaul, some companies have proposed that rewards should simply be complemented by self-regulated commercialization. The proposed regulation was first set-out in the January 2016 antimicrobial resistance (AMR) Industry Declaration [8] at the World Economic Forum in Davos (Davos Declaration). There are, however, few actual commitments for antibiotic conservation and those that exist are somewhat vague and largely focused on other stakeholders. For example, the role of promotional activities is not referred to at all and perverse incentives (where remuneration can promote unit sales) are only addressed with respect to other health-system stakeholders, not the remuneration practices of drug companies. The later Industry Roadmap on AMR [9] went further in addressing conservation issues and in one case, made a specific, time-bound commitment. However, the lack of accord or overlap between these two documents with respect to product conservation and the fact that only 13 of the 99 Davos Declaration company signatories joined suggests a lack of consensus within industry as to how it considers its own role in antibiotic conservation. Bundling the Davos Declaration and the Roadmap together to report on progress [10] undermines meaningful tracking and prevents the commitments from becoming time-bound, measurable targets. In effect, the commitments do not make industry accountable—which sheds doubt on whether calls for self-regulation should be heeded.

Even if a robust self-regulation framework were to be agreed upon, developed, and independently overseen, the effectiveness of self-regulation in reducing sales is doubtful when the profit incentive remains so strong (and will grow with highly priced new drugs).

This paper explores what could be achieved within the constraints of the pharmaceutical industry’s current modus operandi, yet reaches beyond the apparent simplicity of collaboration or self-regulation. It considers the possibility of contractually-determined conditions for developers accepting large-scale R&D rewards, highlighting design considerations.

## 3. Methods

An extensive mapping exercise comprised of a literature review and expert consultation was used to identify and explore crucial domains that could be targeted to improve sustainable use as part of R&D reward schemes. An initial list of 17 domains was identified, as below:

1. Marketing and promotion
2. Perverse incentives
3. Supply initiation and continuity
4. Surveillance—data collection and disclosure (sales and emerging resistance)
5. Formulary controls
6. Post-market (clinical) data generation
7. Environment (supply chain pollution)
8. Non-human use
9. Third-party control of the product
10. Availability challenges
11. Sub-optimal clinical use
12. Supply chain challenges and risks
13. Off-label use
14. Substandard/spurious/falsely-labelled/falsified/counterfeit medical products (SSFFC)
15. Market authorization and labelling
16. Pricing and reimbursement
17. Donations

For each of these domains, an extensive review was undertaken, including grey-literature such as policy briefs and WHO guidance documents. This material was used to: (1) explore the current situation with respect to the sustainable use of antibiotics for each issue; (2) find precedent within and across other therapeutic areas; and, (3) identify key opinion leaders, actors, and academics at the forefront of each field.

For each domain, care was taken to get input from an array of stakeholders, including academics, industry, policy makers, and key experts from civil society. Sixty-five individuals were interviewed. Interviews were conducted based on the specific issues pertinent to each domain, but followed a consistent structure based on the following fields of exploration:

- *‘Clear opportunities’ for action*: In optimizing clinically appropriate use. Between the current situation (where we are now) and the desired situation (where we would like to be).
- *No likely lego-regulatory response*: Anticipated to address the issue in the next decade. The likely absence of a legislative or other system-wide mechanisms increases the imperative to tie the issue to a specific R&D incentive.
- *Reasonable industry influence*: Over the issue given their role in the antibiotic lifecycle (product development, production, and global sales and distribution). For each domain, do developers have ‘reasonable’ responsibility or influence? To what degree could a condition be considered reasonable and practical bearing in mind the comparative advantage and cost relative to other stakeholders who could potentially take on a greater role in sustainable use in this space.
- *Realistic to address early in product development*: Are the risks presented by the identified domain largely general to the high unmet need antibiotic class and the general pharmaceutical business model? Are they conceivable and hence could they be, at least, mitigated through an early-defined condition that would be potentially blunt and non-specific but would maximize early developer certainty.

Regarding these fields of investigation, it is very much anticipated that to optimally mitigate SU risks, a second-wave of ‘late-stage’ conditions, i.e., perhaps distribution controls, named-patient supply systems, strengthened third-party oversight, may be necessary towards the end of a candidate’s development (as a novel antibiotic approaches dossier submission for marketing approval). This is when the true characteristics of the specific candidate product and the ultimate launch and policy environment will be much clearer. This research focused on the former more general and conceivable risks that could, at least in part, be addressed early.

Domains (1–9) were categorized—and are henceforth listed in order of decreasing priority—as those for which an SU-related condition on developers was perceived to be necessary and reasonable (affirmative for all of the above-mentioned criteria). The remaining domains (10–17) were excluded from further detailed exploration, because the provisional analysis revealed negative, uncertain, or non-affirmative answers to one or more of the above-criteria.

*Sustainable use* (SU) is the expression used henceforth to cover the principle of antibiotic conservation and stewardship occurring in the long-term.

## 4. Findings and Discussion 

The findings presented below represent general domains (or clauses in a contract with a public body), which could be clarified to developers and reward participants—early-on—in novel antibiotic development. They have been presented in order of the perceived importance of the proposed developer conditions for achieving overall sustainable use goals. Further conditions will likely need to be determined, in negotiation with all parties (closer in time to market authorization), depending on a product-specific risk assessment (premature efficacy waning, cross-resistance profile, etc.) and country-specific lego-regulatory contexts (if sufficient sustainable use-related safeguards have been implemented by national governments).

## 5. Domain 1—Marketing and Promotion

### 5.1. Background and Justification for Inclusion in Developer Conditions

The marketing and promotion of antibiotics in the traditional unit sale-based business model is used to maximize sales, with the aim of recouping the high costs of R&D investment and seeking profit. In this sense, the magnitude of the promotional activity and intended sales is largely detached from the justified clinical need for the product. Overall, given the strong incentives to promote antibiotic sales and the corrosive effects that high-volume consumption of antibiotics has on product efficacy, decisive efforts to curb antibiotic promotion are warranted.

Governance of pharmaceutical company communication practices in high-income countries (HICs) utilizes an array of legislative, regulatory, and voluntary or code-based compliance control mechanisms [11]. In many low- and middle-income countries (LMICs), any form of regulation remains weak, non-existent, or poorly enforced. A 2004 study by WHO [12] showed that fewer than half (49%) of the world’s countries had introduced any legislative framework to regulate the promotional activities of the pharmaceutical industry and a number of World Health Assembly Resolutions have urged member states to do more [13]. Worldwide, certain specific practices are often illegal and these can include: false, misleading, and fraudulent claims; direct-to-consumer advertising; and off-label promotion (dissemination of information inconsistent with their products approved drug labels or ‘intended uses’). These systems display much discussed inadequacies in ensuring medicines are promoted accurately, ethically, and to the appropriate degree.

In most countries, in previous decades, pharmaceutical marketing activities were more explicit. Printed materials and face-to-face time between company representatives and prescribers, in their clinics or at conferences, formed the mainstay of this communication interface. As many of these activities were curbed through regulation [14] and with the advent of digitalization, prescribers’ home computers and smartphones increasingly represent the new interface. The vehicles used to propagate messages to, and influence, physicians have burgeoned and now include; email, search engine optimization, use of social media, peer-influence, key opinion leaders, and even continuing medical education fora [15]. In the United States, a regulatory shift was seen in 2009, to focus on the quality and disclosure of the underlying data, away from the circumstances of the dissemination itself. However, given today’s new tools, it may be that the data and messages are so dispersed and transient as to be inaccessible to regulators.

These shifts have two major implications. First, differentiating legitimate healthcare professional education from promotion is now considerably more challenging. The conflict of interest that exists when pharmaceutical companies are involved in the ‘education’ of healthcare professionals has been highlighted previously [16]. More recently, and specifically with respect to antibiotic stewardship, the independent AMR Benchmark indicated that the line between these education and stewardship goals if often blurred. Companies generally lack clear educational targets, use similar content and goals for both marketing and educational purposes, and some programs are reported as having both marketing and educational purposes [4]. This opacity in the source and integrity of the communication messages poses a challenge to both regulators and the healthcare professionals themselves.

The second challenge is that existing lego-regulatory tools for addressing concerns in this field are becoming redundant. As they do so, they are leaving ever-widening gaps in how companies are able to exert undue influence over prescribers. This is something that the FDA has largely acknowledged with its 2009 public hearing on the use of internet and social media for promotion [17]. As with broader internet governance discussions, regulators are wrangling with how to address the complexity of new pharmaceutical marketing realities. For the moment, lego-regulatory oversight remains focused on the messaging used in, largely, printed materials, which likely form a tiny proportion of the ways that companies now reach healthcare professionals. Additionally, no regulator has a specific mandate with respect to antimicrobial resistance, i.e., to ensure that pharmaceutical messaging is aligned with ’stewardship goals’. For the moment, as with other therapeutic classes, they look at whether messages are truthful, balanced, and accurately communicated, but this mandate is likely insufficient for when a product is not needed or when good antibiotic stewardship would suggest the use of a different product—both potentially contributing to the growth of resistance. Attempts at strengthening the role of regulators with respect to antibiotic promotion, such as the additional labelling and pre-review requirements of the FDA’s office of prescription drug promotion (OPDP) Limited-population Antibacterial Drugs (LPAD) scheme, are likely too weak to address the specific precautions with respect to prescribing that which stewardship would demand.

In areas of the world largely dependent on self-regulation and in the absence of effective enforcement and monitoring, parties to such voluntary schemes have an incentive to adhere only to the extent that they deem it in their interest to do so [18]. When the marginal sale of a novel antibiotic is likely to be so profitable, it remains difficult to see how it would be in companies’ interest to proactively address all but the most glaring breaches of stewardship goals. Early experience with companies’ voluntary action in this area [8] over the past two years does not reveal much concrete or verifiable action. The absence of robust and specific regulatory tools to address the challenge that excessive or unbalanced promotion may cause to SU, places a greater onus on the need for decentralized oversight. Decentralized accountability processes can be significantly strengthened by the addition of a whistle-blower function. Whistleblower actions have been remarkably effective at bringing to light more problematic and less visible company actions. The whistleblower case surrounding off-label marketing of tobramycin by Novartis (with a settlement of $US 72.5 million in 2010 [19]) is one example. Indeed, approximately 90% of health care fraud cases are qui tam actions in which a whistle-blower with direct knowledge of the alleged fraud initiates litigation on the government’s behalf [20] and benefits through receipt of some or all of any penalty imposed by the prosecution. The problem lies much deeper than the mass promotion of antibiotics that arises through the propagation of misleading messages. It stems from the undue influence that companies derive from a growing plethora of communication channels to sway prescribers to prescribe their products (e.g., sponsored events, conferences, email, search engine optimization, use of social media, etc.), including in cases where they are not needed or where it goes against good stewardship practice.

### 5.2. Considerations for Condition Design

**Figure 1:**
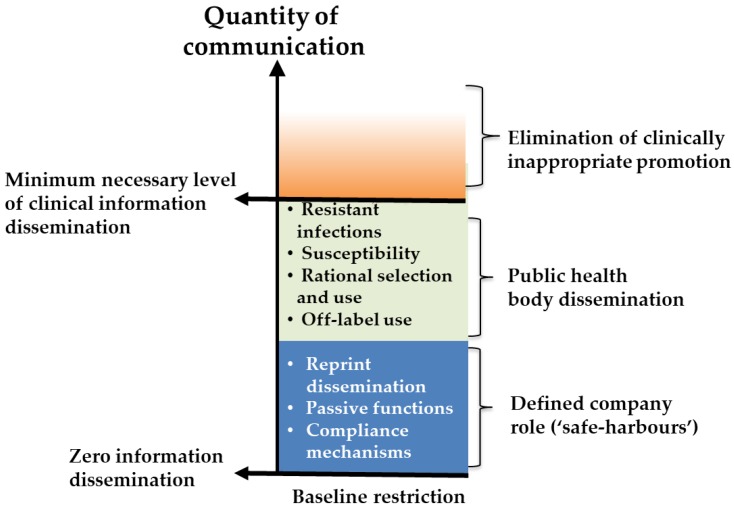
Conceptual representation of proposal for defining more appropriate boundaries for company marketing and promotion of a novel antibiotic.

Any attempt to curb product promotion must ensure that minimum essential information about product usage and safety is relayed as appropriate to prescribers, whilst eliminating clinically inappropriate communication (Figure 1). Given the fine line between essential information exchange and product promotion, a greater role for alternative actors, such as health authorities, should be considered. Indeed, a number of proposals have been put forward in this regard. For example, a multi-stakeholder approach to communications with health care professionals has been suggested [21], as well as the expanded use of ‘academic detailing’ techniques, such as those that have been developed and championed in the United States and used increasingly to tackle the explosive use of opiates [22]. However, while shifting the responsibility to the hands of impartial public health actors is desirable, it may prove impractical to some degree given their limited resources and lack of expertise in direct-to-physician information exchange (especially on the scale necessary for widely used products)—at least in the short-term. Although, given the huge sums that companies spend (and then must recoup through sales) on the marketing of a new product, removing or diminishing the developer’s role here could justify a resource shift to accompany this task-shifting from the private developer to public health authorities. While the authors acknowledge that presently public health authorities are largely unprepared for such a role, it is clear that increased financial support to build such capacity could come, at least in part, from reward savings.

The use of off-label legislation could be usefully applied to on-label usage of any novel antibiotic reaching the market through a publicly-subsidized pull-incentive to curb product promotion. While many countries ban the promotion of products for uses that are not licensed (those not covered by the marketing authorization or the label), applying the same legislation to achieve a baseline ban on promotion of on-label use of novel antibiotics would have substantial advantages.

For the last 25 years, the US FDA has been publishing guidance to industry to clarify what is legally permitted and what is not [23]. This provides a wealth of policy experience in determining boundaries between what is and what is not acceptable promotion and it serves as a useful reference for defining ‘safe-harbors’ in which companies would be permitted to communicate with healthcare professionals about their products if the circumstances, means, and messages were appropriately aligned with stewardship goals. Starting with a baseline ban on product communication (as with the off-label experience) and building up, it is practical in that companies are very familiar with off-label legislation and the limits that it sets. It could also make use of the existing legislative apparatus, which has an immense global reach, and thereby support the regulatory infrastructure in countries with weaker systems.

Proposed condition: No product promotion, as per existing off-label restrictions, with defined exceptions (‘safe-harbors’) for the dissemination of use-related information.

- To be supported by an enhanced role of public health bodies in informing practitioners etc.
- A globally accessible, anonymous, whistleblower facility (supported by a policy of non-retaliation and by qui tam style financial incentives).

## 6. Domain 2—Perverse Incentives 

### 6.1. Background and Justification for Inclusion as a Developer Condition

While some perverse incentives inappropriately affecting antibiotic sales arise from within the health system itself (and are the responsibility of government)—such as when prescribers are able to benefit financially from the prescription of an antibiotic [24]—others are a direct result of company behavior. For example, information asymmetries and a company’s financial leverage can result in influence over employees, healthcare managers, and healthcare staff (including prescribers) that can undermine clinically optimal practice and directly encourage imprudent use of the antibiotic (see below for examples).

Such practice is often governed by a network of guidelines, codes, and laws that exist at multiple levels. Although most current legislation mainly applies in countries with ‘strong lego-regulatory systems’ (i.e. HICs, and in the last decade, also some upper middle-income countries, such as Russia, China, Brazil, and South Africa, which all have legislation in place). Outside of these countries, the WHO has had the Good Governance for Medicines (GGM) program [25] in place since 2004. Its goal is to contribute to strengthening heath systems and reducing corruption in pharmaceutical systems through the application of transparent, accountable administrative procedures and the promotion of ethical practices. As of the end of 2012, less than 20% (36 of 192) of countries and territories were engaged in the GGM program [26].

To take one example of how these issues are addressed at a national level, the US has provisions for perverse incentives within criminal law (prohibiting bribes); licensure laws (prohibiting fee-splitting); formulary-specific laws; and other regulatory prohibitions, such as stark or anti-kickback statutes (AKS) when occurring domestically and the Foreign Corrupt Practices Act (FCPA) when occurring abroad. Despite the Security and Exchange Commission (SEC)’s acknowledgement that ‘bribes come in all shapes and sizes’, three types of misconduct were highlighted in a 2016 report [27] as being the most prevalent: pay-to-prescribe practices, bribes to ensure drug approval or formulary inclusion, and bribes (including discounting mechanisms and rebates) disguised as charitable donations. All of these practices would significantly undermine sustainable use efforts if undertaken for a novel antibiotic. Another area that has garnered much attention in recent years, resulting in commitments by many large multinationals (AstraZeneca, Bayer, Eisai, Eli Lilly, GSK, Merck KGaA, and Novartis) [28], is sales-based incentive targets for their sales forces and the impact these have on prescribing practices. As of 2018, two companies have remained true to the commitments and decoupled remuneration from the volume of sales [4].

### 6.2. Consideration for Condition Design

The combination of weak or non-existent legislation in many countries, along with insufficiently comprehensive and poorly followed attempts at industry self-regulation, suggest that sales and distribution-related perverse incentives could be addressed within product-specific conditions placed on developers. Because these incentives occur within complex sales and distribution systems that change over time, broad and general wording is necessary and is needed to make the conditions time-resilient. This approach ensures that a condition may function as an umbrella that can apply even as the specific techniques and tools shift and evolve over time. Practices that may result in inappropriate registration, formulary inclusion, reimbursement, or prescription inducement are not only diverse, but also tend to evolve in response to changes in the health system and lego-regulatory environment. Challenges in determining lines between appropriate and inappropriate practice may also prove challenging, for example, in the area of rebates (price discounts based on volume) and kick-backs (where individuals benefit). This will require further exploration by legal professionals.

Proposed condition: Identification and elimination of inducements that may encourage clinically unjustified use of the product, including (but not limited to) volume-based remuneration of staff and payments/benefits-in-kind to prescribers.

## 7. Domain 3—Supply Initiation and Continuity 

### 7.1. Background and Justification for Inclusion as a Developer Condition

Supply commitments of developers cover two areas considered ‘risks for [un]sustainable use’:

- *Minimum national safeguards*: the risk to waning product efficacy from being used in countries without minimum product-specific SU safeguards in place (risk of oversupply where there is an insufficient ability to provide the product in an appropriate manner).
- *Supply continuity*: the risk to appropriate stewardship from disruptions in the supply of the novel product (risk of undersupply when the product is in routine use).

We address these separately below, although they are eventually brought together in a single condition.

### 7.2. Supply Initiation and SU-Safeguards

Oversupply or over-consumption (beyond that which is clinically necessary) of a novel antibiotic risks amplifying or expediting the spread of resistance, globally. When considering antibiotic efficacy as a global public good, there is sense in maintaining some control over its distribution and use. At the national level, making supply of the novel product conditional on each country having signed a declaration that they meet a pre-specified minimum list of SU safeguards could serve as the carrot to ensure that minimum SU provisions are in place with the requisite political and administrative support for each country needing the product. Practically, this could be achieved in a number of ways and will largely depend on the administrative capacity behind the rewarding entity. At one end of the spectrum, this function could remain with the supplying company. They would hold responsibility, via a contractual condition, for deciding which markets to supply (i.e. companies would have to contractually accept to only supply the product to countries having declared to have the specified SU provisions in place). However, this may be undesirable or unethical for a number of reasons, not least because this would amount to outsourcing a national government accountability function to a private actor (with its inherent conflicts of interest). The economic incentives suggest that companies would have a stronger incentive to increase the number of countries through who sales can be generated than to withhold supply if safeguards were insufficiently assured. More practically, it may be that companies might not have the expertise to perform the necessary monitoring and oversight functions and would be unable to provide technical assistance to countries to help them achieve the necessary minimum safeguards.

An alternative approach, which has many merits, would be for the responsibility for certification/management and/or conditional distribution (‘green light’ function) to lie with a ‘broker; either the reward administration entity or a delegated third-party. The global roll out of bedaqualine for multidrug resistant (MDR) tuberculosis (TB) provides precedent both for how this green-light role can be arranged and how rational-use can also be achieved in low-income country (LIC) settings. Countries meeting minimum WHO requirements, as determined by the Green Light Committee, were eligible to receive ‘concessionary prices’ for the product through the Stop TB partnership’s Global Drug Facility [29]. It may not even be necessary to replicate such an arrangement, but merely to expand those that exist.

### 7.3. Supply Continuity

The last decade has seen increasing attention given to global supply shortages of medicines [30]. While the bulk of the risk arises in the generic (off-patent) market, shortages are also increasingly seen in branded medicine markets, including in HICs [31]. National shortage tracking systems are being put in place (examples US and Australia) and these reveal a growth in the scale of the problem since 2007/2009. Antibiotics are one of the therapeutic areas most affected, with one study reporting that 22% of antibacterials suffer from multiple shortage periods [32]. Shortages also impact antibiotics to treat MDR-infections, with 46% of shortages impacting the treatment of high-risk pathogens. WHO has been actively working on the issue, most recently with the World Health Assembly Resolution on the topic in May 2016 [33].

Shortages of antibiotics pose particular problems for stewardship. While supply disruptions can happen for a multitude of reasons, greater responsibility must be taken and more effort is needed to overcome what is, in some cases, the formidable technical challenge of maintaining continuous high-quality antibiotic supply. Due to the complexity of supply networks, this is an area where multiple stakeholders ultimately influence supply; however, the role of manufacturers is pivotal due to the agency relationship they have with their active pharmaceutical ingredient (API) suppliers.

We argue that a condition is likely needed for a novel antibiotic to fulfil an unmet medical need to ensure that we move beyond mere ‘best efforts’ to more concrete action to prevent antibiotic shortages. However, the nature of the condition requires further exploration and, indeed, could bind manufacturers to a target or goal while leaving flexibility for how they address this both within the parameters of their responsibility (or influence) and according to their unique expertise and supply relationships. For example, they could be asked to submit a supply continuity plan outlining how they intend to achieve the goal through the use of tools, such as rotating stockpiles and a diversity of API manufacturers. A rotating stockpile can help to ensure that a sufficient stock of products is available and accessible to countries at short notice. The benefits of the long-term supply agreements to the end-product and API manufacturers can facilitate competition by guaranteeing a regular stream of orders. So, when external supply disruptions occur, the stockpile can be used to fulfil orders in the short-term. Again, this is a tool presently used for the global supply of TB products. Experience with products such as HIV and TB has revealed that diversity of API suppliers/production sites can also help to mitigate some supply disruptions; there is some consensus that having at least three producers or production sites is essential to ensuring supply continuity. Others have indicated some value in bringing some production closer to the end-product manufacture, for example, through inshoring or nearshoring [34] core product components. A plan highlighting how a company would employ multiple tools and how they would move between them to ensure product continuity would go some way to address this. The plans would vary in their use of the different tools, depending on the final product, the expected demand, the manufacturer, and a host of other factors. Proposed condition: Companies agree not to sell-in to countries or supply channels that either:

- Lack the ability to distribute/prescribe appropriately (unless this is guaranteed by a third-party) and/or
- Do not have an effective national supply control and stewardship plan in place.

Additionally, manufacturers must submit—for external approval—a supply continuity plan to concretely highlight through which means they will honor the supply terms of agreements with countries in a timely manner.

## 8. Domain 4—Surveillance—Data Collection and Disclosure (Sales Data and Evidence of Emerging Resistance)

### 8.1. Background and Justification for Inclusion in Developer Condition

WHO highlighted the dearth of country-level antibiotic sales and distribution data in its 2001 Global Containment of the AMR strategy [35]. Public bodies are showing more interest in understanding consumption and resistance trends and more resources are starting to be diverted into this area. On the side of industry, in addition to some post-marketing regulatory requirements which aim to capture some of this information, industry has, for many years, sponsored large-scale surveillance programs which additionally aim to inform commercial strategies [36]. However, the overall picture remains patchy, incomparable, and disproportionately available from richer countries.

Regarding consumption, we are likely to remain dependent on triangulating imperfect data from multiple sources/actors at different points along the supply chain to piece-together a picture still partly based on extrapolation and estimation for some time. However, this picture could be improved were data (price and stock levels at each level in the supply chain, including end-user demand or consumption) to be collected from both sides: from within the health system itself and from suppliers/manufacturers (through product tracing). This dual system would help overcome the conflict of interest in relying on industry-provided data.

Indeed, companies themselves already collect vast amounts of data on where medicines are sold and used. Some of this data is collected as part of the post-market surveillance obligations of regulators, i.e., estimation of patient exposure (including all data relating to the volume of sales and prescriptions) is a reporting requirement of EMA’s periodic safety update reports (PSURs) [37], but all of this information has great commercial value to the companies in understanding where sales are generated and from where product demand originates. Additionally, companies claim great interest in supporting public surveillance efforts [9]. However, at present, two challenges prevail with this data. The first relates to quality, as sales data (data on price and unit volumes) is a relatively poor proxy of use (largely due to the complexity of the pharmaceutical supply chain). The second relates to comparability as companies often use different units and processes than those used by the public sector. Regarding the former, the advent of track-and-trace (traceability) legislation aiming to address supply chain deficits with respect to security and product recalls perhaps presents some hope that issues (of knowing where specifically a product ends up and when it is consumed) may be resolving. Both the EU Falsified medicines Directive (EU-FMD) [38,39] and the US Drug Supply Chain Security Act (US-DSCSA) [40] legislation require that serialization numbers and bar codes be added to the smallest saleable pack of each medicine, and either bookend tracking or complete end-to-end tracking (until a product is decommissioned: consumed, withdrawn or recalled) be in place by 2019 for the FMD and by 2023 for the DSCSA.

### 8.2. Considerations for Condition Design

Although the technical barriers to manufacturers providing good quality sales data (that can be used as reasonable proxies for consumption) appear to be diminishing, some degree of mutual suspicion on the parts of industry and public health authorities will be harder to surmount. If return on investment is still linked to unit sales, then companies will have an incentive to represent their product in the best possible light (site selectivity, protocol design, comparators or result analysis and disclosure [41], and possible delay or suppression of evidence of resistance development [42]). From the perspective of public health authorities, as long as unit sales remain in place, there is a conflict of interest that taints the quality of any data made available. From the perspective of the company, there can be concerns over how the data will be used once shared. For example, who will have access to the data? This could be addressed through a single body holding the data, granting access on a restricted, ‘need-to-know’ basis. The more substantial concern relates to how the data will be used and whether the data will be used to curtail sales or set a sale/price ceiling. If provision of the sales data could be linked to punitive or other actions that could impact the commercial situation of a company, then we are unlikely to see any movement or de-proprietarization of this data anytime soon.

Interviews conducted with surveillance specialists as part of this study indicated that the value of companies providing accurate data on what antibiotic products are entering each medical facility (certainly each hospital) was considered desirable and helpful for public health efforts to better understand consumption patterns and trends. Interviews revealed anatomical therapeutic chemical (ATC) and defined daily dose (DDD) to be the most useful units of reporting in order to enable conversion and maximum flexibility in data processing. Within low- and middle-income countries (LMICs), where gathering such data would arguably be more valuable for low-priced, generic products, starting with originator manufacturers and the highest-need novel products may help set industry standards in the area.

Proposed condition (part A): Company will make the best efforts to contribute data and financial resources in the creation of a harmonized, fully-transparent, and publicly-led AMR surveillance system. However, in the more immediate term, it will specifically conduct time-bound collection and reporting (as packs and ATC-DDDs) of product volumes—adjusted for redistribution—by country and supply-channel and healthcare-facility-level (as feasible and relevant to the country context).

With respect to the health system surveillance of resistance development and trends, there has been significant progress in recent years surrounding standardization, including through efforts of the European Antimicrobial Resistance Surveillance Network (EARS-Net) [43] in the European Union and WHO’s AMC network. However, while improving, these have fallen short in some respects, possibly due to the reliance on voluntary reporting and challenges in achieving standardized collection by member states [43], and the absence of a rapid signal detection system for resistance to newly introduced agents. From the company side, there is not presently an explicit condition to specifically monitor and report data on resistance (emergence and trends), although this data may be voluntarily reported if it becomes a safety issue or the efficacy becomes substantially impacted [44]. However, concerns over companies fulfilling post-market obligations have been raised in recent years.

The situation leaves us belatedly using fragments of data to gauge the resistance situation. Fortunately, however, we are seeing some very encouraging interest amongst companies to move towards the use of common protocols that will help increase the quality and usefulness of the resulting data. We argue here that until robust systems are reliably available worldwide to monitor resistance as soon as it develops, all actors with a stake in the issue should have a responsibility to report and share knowledge of any cases of emerging resistance they become aware of in the years immediately following launch. However, until this is truly enforced, contract-based conditions can help ensure compliance.

Proposed condition (part B): AND if company becomes aware of cases of emerging resistance, it will rapidly inform relevant national authorities (including ministry of health, medicine regulator and focal point for emerging public health threats).

## 9. Domain 5—Formulary Controls 

### 9.1. Background and Justification for Inclusion in Developer Condition

Medicine selection is often a two-step process, which precedes and guides rational use. The first step is medicine licensure, whereby national marketing authorization requires that standards are met with regard to a product’s safety, efficacy, and quality profile. The second step, medicine list (ML) inclusion, seeks a comparative assessment of that product’s value relative to other medicines within a therapeutic class [45]. Medicine lists or formularies are an index, or list, of pharmaceuticals approved for prescription by practitioners. MLs can vary in their level of influence from legally binding tools (e.g., Germany) to guidelines aimed to encourage one prescribing decision over another. WHO defines MLs and clinical guidelines as two of its 12 key tools in promoting the rational use of medicines [46]. Generally, formulary controls are valuable in improving prescribing quality and exerting an influence over certain medical interventions (both products and services) [47]. Formularies can exist at multiple-levels; at the global level, this is the WHO Essential Medicines List (WHO EML), which can be seen as a ‘model list’ or template that is often adapted by national/local authorities to reflect local realities. ML’s can exist at different levels within a health system, i.e., at a national-level, at a hospital-level or, in multi-payor systems, developed for separate payors.

Three important changes to the WHO’s approach to the EML over the past two decades make it ever more relevant for SU for novel antibiotics. The 2002 addition of several high-priced HIV medicines to the list reflected the importance of these products to health systems’ ability to meet their populations’ needs. The second occurred in 2006, with WHO strongly recommending the inclusion of oseltamivir (Tamiflu^®^) for Avian Influenza, which showed that products can be included on the list even while evidence is still pending—which will undoubtedly be the case for novel antibiotics that are expedited to market in the case of a high unmet medical need and few therapeutic substitutes. Finally, the publication of the 20th edition of the EML in 2017 included a revised approach to the antibiotic classification to “ensure [patients] get the right [antibiotic], so that the problem of resistance doesn’t get worse”—making the EMLs role in supporting sustainability ever more explicit. Indeed, the three-tiered ‘risk-based’ classification of antibiotics (access, watch, reserve) [48] is now linked to specific recommendations on their availability, selection, and use.

When combined, these changes and characteristics mean that the WHO’s EML is increasingly likely to remain as, and serve as, the conduit not only vital for expanding access, but also for expanding and supporting globally-coordinated SU efforts. In fact, WHO’s EML may prove vital not only in embedding minimum SU considerations globally, but providing the vehicle on which any necessary ‘late-stage’, i.e., after a product has received its first registration, SU provisions can be built in a nationally appropriate and product-specific way. Examples of how these formulary measures can be strengthened in a product-specific way include combining them with permission requests (with or without approval by certified ID physicians or clinical pharmacists after 48 or 72 h of use); non-reimbursement for products not on the list; and compliance measures, such as audit and feedback of prescribing practices [49]. Even without these additional measures, the risk-based classification of products will be essential to help support countries in adopting policies that balance immediate public health needs with future risk of resistance. Many countries now have multiple National ML’s, one for each health system tier (e.g., India and South Africa), which adds additional power to the formulary by ensuring that the product is only available at the level in the system with appropriate prescribing and diagnostic capacities [50].

### 9.2. Considerations for Obligation Design

Encouraging developers of antibiotics to proactively put dossiers of their product to WHO for potential inclusion in the EML would have many SU-related advantages, particularly with regards to LMICs. Although originator products do feature extensively on the list, currently, only a minority of EML submissions are initiated directly from developers [51], with most being initiated by WHO itself, civil society, or professional groups. Historically, developer caution and push-back against the EML concept has been strong for fear of being excluded: ‘’No manufacturer wanted to have the suggestion that their medicine was less essential, less necessary than others [52]”. Despite its potential role in expanding access (and therefore sales), skepticism may remain over the access expectations that a listing may create for developers, particularly with respect to pricing, national registration, and availability. However, at the point of product launch, developers have by far the best understanding of the product and as such are best placed to submit the required evidence and engage in a dialogue with the EML team about its potential. Indeed, the submission process itself may act as an important link between developers and the countries who will ultimately steward the use of their product.

Proposed condition: Developer will seek a timely dialogue with the World Health Organization (WHO) to explore inclusion of the novel antibiotic on its Essential Medicine List.

## 10. Domain 6—Post-Market (Clinical) Data Generation 

### 10.1. Background and Justification for Inclusion in Developer 

The responsibility for the stewardship of antibiotics is largely acknowledged to fall on the health systems in which these products are used. As laid out in the WHO’s Global Action Plan, a variety of measures will need to be employed by national policy makers. Existing national procedures and practices that determine appropriate uptake and utilization will likely need to be revised to align them with stewardship goals. As with all areas of policy making, this will require an evidence base prior to implementation. Furthermore, new antibiotics will be granted market authorization based on limited evidence of safety and efficacy, which is justified for novel products due to the dearth of therapeutic alternatives. However, this gap between the data available at the time of licensure, and the likely higher (or more rapid) evidence requirements to support that stewardship policies are in place from the outset, presents a challenge from an SU perspective.

First, knowledge of a product’s specific resistance profile takes time to reveal itself. Because the product’s resistance attributes are a minimal and diminishing focus of regulatory agencies [53], it follows that these characteristics are not investigated by developers prior to approval. This means that knowledge of resistance and cross-resistance challenges often emerges later than is optimal, particularly for the proactive (or ex-ante) justification of timely controls. Second, if the burden of collecting further clinical evidence to support appropriate uptake and use remains unspecified within the (R&D incentive) reward criteria, then it will fall on public bodies to collect this data—thereby imposing a ‘second wave of public cost’ in addition to the reward payment. Finally, for a normal medicine being developed under the traditional pharmaceutical business model, the incentive to generate the extra data required for optimal product adoption and uptake aligns with commercial goals. However, in the case of antibiotics, those commercial levers may be dampened and hence companies may require extra encouragement to generate and share the data.

Currently, policy makers either scrabble around ex-post to build a sufficient evidence base to prove ‘scientific consensus’, thereby only addressing resistance after it is already developing, or fight to use the ‘precautionary principle’ [54], as the WHO has done within the Global Action Plan which enables ‘preventative action based on evidence of a ‘plausible risk’—but this mechanism is unlikely to be available to policy makers. Additionally, previous products that have been subject to product-specific stewardship measures, such as bedaqualine for TB, were implemented due to serious, poorly-understood safety concerns over the product itself [55] and not primarily due to concerns over resistance development.

### 10.2. Considerations for Condition Design

In order to avoid a second wave of public expenses and to ensure that companies are optimally supporting national stewardship (as they state in their industry commitments), a condition to generate and share vital clinical and safety data—at least with public health authorities—is merited.

This could be achieved by using a contractual condition such as that proposed below. However, to be effective, this condition would have the role of supporting the conditions laid out in the reward criteria itself. A target product profile (TPP) is likely to form the part of the reward criteria that pertains specifically to the product itself. The TPP lists the minimal technical attributes of an optimal product and spells-out exactly what, approved, product would be eligible to receive a public reward. Additionally, in the situation of public subsidy, the TPP development process facilitates stakeholder alignment of a product prior to its development and directs a developer to undertake an efficient clinical development plan (CDP).

An ideal incentive would ensure the optimal alignment of the TPP with the CDP, with the product label (legal parameters of the indicated or approved uses), and ultimately the product’s clinical uptake and use. However, to impact the latter, the TPP would have to be broader than is typical, perhaps stating minimum affordability, indication, and resistance attributes. Precedent exists for such post-market considerations being included within TPP’s. For example, for some products developed through public-private partnerships (PPP’s) to meet unmet medical needs in LMICs, the price (affordability) of the product is considered such a fundamental component of appropriateness for its target population that this is specified to the developer in the TPP [56]. WHO’s TPP’s for vaccines include ‘programmatic suitability’ characteristics, such as thermo-stability, maximum packed volume, and considerations of preparation for administration requirements [57]. By broadening the reward criteria (making this data a precondition of the reward itself) and placing a time-limitation on data generation and availability, the company could be directly tasked with facilitating the product’s timely adoption, uptake, and—importantly for SU—optimal use.

Proposed condition: Work collaboratively with public health authorities to develop a robust and time-bound approach to capture clinical data—including comparative data—that will facilitate timely fulfilment of the full breadth of the reward criteria, including those pertaining to post-market requirements.

## 11. Domain 7—Environment (Supply Chain Pollution)

### 11.1. Background and Rationale for Condition

Evidence has been mounting that high concentrations of antibiotics have been found around manufacturing sites, largely through the release of wastewater. This represents a potential source of antibiotic resistance development and spread, which companies have the tools and ability to mitigate. Improved production standards and minimized release of antimicrobials into the environment throughout their life cycle (API development, finished product production, use, and disposal) would be reasonable expectations of any developer receiving a reward. All stakeholders acknowledge the problem and work is underway to address the knowledge gaps.

The EMA is expected to strengthen its Environmental Risk Reporting requirements and the European Commission itself has for some time been working on its Pharmaceuticals in the Environment (PiE) legislation. As the likely passing of this legislation has neared, industry’s response has increased in maturity. While largely absent from the Industry Declaration in 2016, the environmental commitments made by selected companies in the 2016 Roadmap were built on in 2018 with the publishing of a specific declaration on this aspect of AMR in its Antibiotic Manufacturing Framework [58].

### 11.2. Contractual Condition

The issue of environmental impacts of AMR is a global and sector-wide issue and as such, placing a condition on a single (or perhaps handful) of developers of novel antibiotics is an imperfect response. One suggestion—strongly opposed by industry [59]—is to include environmental controls (including standards for waste management) in the existing global Good Manufacturing Practice (GMP) framework. GMP legislation is widely in place around the world and provides minimum quality and consistency requirements for all manufacturers of pharmaceuticals. However, this seems an unlikely lego-regulatory development in the short-term. At the same time, self-regulation by industry is likely to be insufficient due to the substantial conflict of interest that exists here (putting the controls in place, improving transparency and disclosure processes, and addressing pollution incidents when they occur are all costly for developers). Opponents also point out that companies may lack the required knowledge and expertise to genuinely tackle this. The low starting baseline of the sector, two years after the commitments were made, was highlighted in the 2018 AMR benchmark, which found that currently only eight companies have discharge limits for antibiotics (with none disclosing the levels) and only one company names its third-party manufacturers.

However, there is evidence emerging that some companies are not just implementing best practice in their own manufacturing sites, but are also facilitating the environmental risk management of their suppliers. A condition could be justified as a useful catalyst to these efforts to mandate, at least, current best-practice from any reward recipient, as the arbiter of what would be a new generation of antibiotic products and perhaps as a stop-gap until effective sector-wide and global regulation is able to mitigate these risks.

Proposed condition: In-line with best practice in the sector at the time of reward disbursement, the reward recipient must make all actors in the supply chain of the product transparent. Furthermore, they must ensure their—and those of their suppliers—discharge limits remain below predicted no-effect concentrations (PNEC) for resistance selection and that breaches of environmental risks are effectively mitigated for and disclosed.

## 12. Domain 8—Non-Human Use

### 12.1. Background and Justification for Inclusion in Developer Condition

Consumption of antibiotics is common in both human and animals. Indeed, the majority of antibiotic use occurs in animals for reasons of disease prevention, treatment, and growth promotion. Crucially, the combined use across humans and animals includes several critically important antibiotics (CIA) that are considered essential for human health (Quinolones, 3rd and higher generation Cephalosporins, Macrolides and Ketolides, Glycopeptides, and Polymyxins [60]). Use of such medicines in animals is increasingly worrying given the substantial and expanding volume of evidence reporting animal-to-human spread of resistant bacteria.

This risk of efficacy erosion due to use in animals is—and has for some time—been acknowledged. Importantly, it has been recognised by the World Organization for Animal Health (OIE), which has been a proponent of a prudent approach and cognizant of its role with respect to human medicine [61]. For example: ‘when a new class of drug comes on the market, it should be considered critically important from the outset unless strong evidence suggests otherwise’ [62]. The Codex Alimentarius Commission (CAC), active since the 1960s, governing global food standards and trade, issued a code of practice, in 2005, to minimize and contain AMR [63]. Since 2003, a tripartite commission comprising the Food and Agriculture Organization of the United Nations (FAO)/OIE/WHO has been working to coordinate recommendations across sectors.

At the national-level, stringent regulatory authorities (SRAs) licensing new medicines for use in animals already take into consideration human health safety risks, although through differing approaches [64]. Additionally, unlike marketing authorization for human use, animal medicine regulators already have the mandate to initiate regulatory action (Table 1) based on resistance concerns.

### 12.2. Considerations for Condition Design

It is clear that limiting the use of a new antibiotic product to human medicine would be advantageous for reducing selection pressure and ultimately slowing the growth of pathogen resistance. Also, in the short- to medium-term, the need to use a high need novel product in animals is unlikely. Furthermore, in HICs, animal regulatory authorities already have the mandate, processes, and tools in place to assess and act on these risks of cross-species transmission. However, as revealed by a 2005 OIE survey of its member states [68], other areas of the world cannot depend on sufficient regulatory competence or enforcement capacity to support appropriate stratification and risk-mitigation of products across sectors. While the work of the tripartite commission goes some way to support the implementation of these measures in LMICs, very large policy differences persist in different regions of the world [69]. This is in part due to the fact that in practice, their recommendations have little weight against the strong agricultural interests in many middle-income countries (MICs). Given these weaknesses—and the longer-term uncertainty—placing a restriction on manufacturers as an interim measure may help mitigate the risk of inappropriate use arising from animal use of this novel product. 

In theory, developers have been supportive of such a move; however, in many cases, the products are not considered exactly the same due to adaptations required (reformulations and different routes of administration) due to species differences and the differences in human and animal regulatory requirements. This increases the chance of dual use across humans and animals. Relevant conditions must therefore be carefully worded to avoid the potential abuse resulting from such technicalities.

Proposed condition: Sales of ’active ingredient’ outside of human medicine are prohibited. Should a complete ban prove ethically or politically unfeasible, manufacturers could be asked to develop ‘green’ versions of their novel product prior to animal medicine licensure [70]. These ‘green’ criteria (regarding pharmacokinetic and pharmacodynamics properties of a product) would have to be externally established and verified, and further investigation here is warranted. However, additional steps need to be undertaken by antibiotic developers anyway prior to animal medicine licensure, for example, additional studies (to prove consumer safety in food-producing animals) and reformulations/other product adaptations.

## 13. Domain 9—Third-Party Control of Product 

### 13.1. Background and Justification for Inclusion in Developer Condition

The entity that undertakes R&D is often not the same as the one that ultimately commercializes (introduces and maintains a new product in a market) the end product. Other companies are often involved in these commercialization or downstream activities surrounding manufacturing, registration of the product within country markets, etc. Small and medium enterprises (SME), including biotechnology companies, often do not have much capacity for downstream activities, especially when sales span multiple regions. Indeed, most SME involvement has traditionally been early on or up until market approval, when the product shifts to larger companies with more sales and marketing experience. Large multinational developers also prefer to out-source many of these activities to other companies (partners) if the latter have greater expertise and capacity.

Out-licensing or contracting-based arrangements in pharmaceutical markets normally utilize upfront payments, sales-target-based milestone payments, and/or royalties [71]. In some cases, these can compound the original incentive to sell the additional unit even further, working against SU goals. In addition, there is the risk that because a secondary or tertiary company (licensee) did not directly agree to the provisions, they may be less committed to upholding provisions. This is a particular risk if they are operating in a legally relaxed environment.

### 13.2. Considerations for Condition Design

As SU is largely affected by downstream activities, it is essential that there is a ‘viral provision’ or condition that a reward recipient must pass on all the contract provisions in its contracts to any third-parties/licensees (and they must be subsequently passed on). This may go some way to ensure that SU is embedded or bound to the product itself and not to the initial developer/reward recipient, regardless of how many times it changes hands.

The nature of downstream actors and the environment in which they operate is unknown when the drug is under development. As such, viral provisions are necessary to support SU.

Proposed condition: Licensees/sub-contractors inherit contract terms (Contract terms “follow the product”).

## 14. Conclusions 

Subsidies and larger financial incentives are needed to promote antibiotic development. These provide an opportunity to help enlist the pharmaceutical industry in the fight against AMR. Maximizing profit is the natural goal of the industry and self-regulation is unlikely to promote sustainable antibiotic use. However, contractual conditions tied to public funding can help bring developers on-board with practices that promote public health. The obligations proposed here are considered feasible and concern areas that can be reasonably considered within the developer’s role and capacity. In accepting a large, predominantly public-financed reward, developers of new antibiotics should be requested to adopt business practices that promote long-term antibiotic conservation.

## Figures and Tables

**Table 1:** Examples of possible animal regulatory actions triggered by concerns over resistance in human medicine.

Possible Regulatory Action	Examples of Use
Refuse animal marketing authorization	Fluoroquinolones, Australia EMA tigecycline [65]
Restrict animal marketing authorization	extra-label use of cephalosporins in food-producing animals [66] in the USA
Revise animal marketing authorization	Colistin, EMA [67], introduced restrictions based on new information
